# Change in health status in long-term conditions over a one year period: a cohort survey using patient-reported outcome measures

**DOI:** 10.1186/s12955-014-0123-2

**Published:** 2014-08-12

**Authors:** Michele Peters, Helen Crocker, Sarah Dummett, Crispin Jenkinson, Helen Doll, Ray Fitzpatrick

**Affiliations:** Nuffield Department of Population Health, University of Oxford, Oxford, UK; Oxford Outcomes, Oxford, UK

**Keywords:** Patient-reported outcomes (PROMs), Long-term conditions (LTC), Cohort study, Health change, Responsiveness

## Abstract

**Background:**

Enhancing quality of life for people with long-term conditions by monitoring patient-reported outcome measure scores is a key domain of health care policy. This study investigated the responsiveness of patient-reported outcome measures for long-term conditions.

**Methods:**

A cohort survey was conducted in 33 primary care practices and 4485 patients (1334 asthma, 567 chronic obstructive pulmonary disease, 1121 diabetes, 525 epilepsy, 520 heart failure and 418 stroke) were sent a baseline survey containing a generic (EQ-5D) and a disease-specific measure. Baseline respondents were sent a follow-up after 1 year. Differences in scores for each long-term condition were assessed by paired t-tests. The relationship between scores and self-reported ‘change in health’ was assessed by analysis of variance.

**Results:**

The baseline achieved a 38.4% response rate and the follow-up 71.5%. The only significant difference for the EQ-5D was found for the Visual Analogue Scale in heart failure between baseline and follow-up, and for change in health. Significant differences between baseline and follow-up scores were found on the disease-specific measures for 1 asthma dimension and 1 stroke dimension. No significant differences were found for other conditions. Significant differences between self-reported change in health and the disease-specific measures were found for 4 asthma dimensions and 2 stroke dimensions.

**Conclusions:**

Few significant differences were found between the baseline and follow up or between ‘change in health’ and PROMs scores. This could be explained by the time frame of one year being too short for change to occur or by the PROMs not being responsive enough to change in a primary care sample. The latter is unlikely as the PROMs were in part chosen for their responsiveness to change. The baseline response rates may mean that the sample is not representative, and stable patients may have been more likely to participate. If PROMs are to be used routinely to monitor outcomes in LTCs, further research is needed to maximize response rates, to ensure that the PROMs used are reliable, valid and sensitive enough to detect change and that the time frame for data collection is appropriate.

**Electronic supplementary material:**

The online version of this article (doi:10.1186/s12955-014-0123-2) contains supplementary material, which is available to authorized users.

## Background

All health care systems require methods and measures to track quality of care. Donabedian [1] suggested three dimensions that together constitute quality of care: structure (i.e. the attributes of the setting in which care occurs), process (i.e. giving and receiving care) and outcome (i.e. the effects that care has on health status). In England, the National Health Service (NHS) is increasingly interested in focusing on patient-reported outcomes as indicators for the quality of care [2]. Since 2009, such outcomes have been assessed on a routine basis in four elective surgical procedures (hip replacement, knee replacement, varicose veins surgery and groin hernia repair) by the means of patient-reported outcome measures (PROMs) [3].

An estimated 15.4 million of the population in England report having at least one long-term condition (LTC) and approximately a third report multiple LTCs [4]. A general practice study in Scotland found that 42.2% of patients had one or more morbidities with 23.3% having multiple morbidities [5]. The expected trajectory of LTCs and multimorbidities is a deterioration in quality of life [6,7]. Trajectories can vary in terms of speed of deterioration or periods of improvement and exasperation, but generally little is known about the speed and nature of disease progression in LTCs. People with chronic conditions or limiting LTCs are the most intensive users of the most expensive health care services [8,9] and the majority of health expenditure is for people with chronic conditions [10]. It is believed that the number of those with LTCs, including multimorbidity, will rise in the next few years posing challenges to health care services [11] and leading to additional cost implications [8,12]. Enhancing quality of life for people with LTCs by monitoring PROMs scores is a key domain of health care policy in England [3]. PROMs may also be important tools in outcome assessment in other health care systems.

The role of PROMs in elective surgical procedures is relatively straightforward as they are used to help assess the effectiveness of single, discrete procedures in relation to patients with fairly clearly defined problems for which surgery is normally effective. The role of PROMs in LTCs is far less clearly understood and more challenging to identify. LTCs are complex to manage due to the multiple physical, social and emotional problems they pose and a diverse range of service providers and interventions are involved in their management over long time lines. Often the objectives of health and social care services are to maintain well-being or to avoid deterioration rather than to achieve major health gains. Nonetheless, PROMs may present a method to gain more information on outcomes in LTCs. The specific aim of this article is to report the evidence of whether change in health status occurs after a one year period in a primary care sample of people with LTCs. The assumption that change can be assessed was based on two considerations: first, the NHS Outcomes Framework’s [6] second domain refers to the possibility of changes over time in the quality of life in LTCs and second, the trajectory of many LTCs is a deterioration in health status. One generic and appropriate disease-specific PROMs were selected to assess health status.

## Methods

A postal cohort survey was conducted in 33 primary care practices from September 2010 to April 2012, with the follow-up data being collected one year after the baseline. In total 4485 patients with six LTCs (1334 asthma, 567 COPD, 1121 diabetes, 525 epilepsy, 520 heart failure and 418 stroke patients) were invited into the study. The LTCs were chosen by the Department of Health on the basis of policy relevance and diversity of impacts. The aim of the study was to evaluate the feasibility of collecting PROMs data in LTCs. This article presents the change in health status by means of presenting the change scores between the baseline and follow-up PROMs scores.

Ethics approval had been obtained through the National Research Ethics (NRES) Committee of the Isle of Wight, Portsmouth & South East Hampshire (now the NRES South-Central Committee) in March 2010 and R&D approval from 20 participating Primary Care Trusts (PCTs). The study was registered on the National Institute for Health Research (NIHR) portfolio (UKCRN ID: 8462).

### Design

The baseline survey, together with an information sheet, was mailed from the practices with an accompanying letter from the general practitioner. Respondents returned the baseline survey to the research team. The follow-up survey was mailed from the research team to baseline participants who had given their consent for the follow-up. All surveys were numbered to identify the respondents’ practice and to match baseline and follow-up responses. At baseline, a ‘thank you/reminder’ letter was sent by the practices after two weeks of the initial mailing to all patients invited into the study. The follow-up reminder, also sent after two weeks, was sent by the research team and targeted at non-responders only. A Microsoft Access database was used to manage the mailing and receipt of the surveys. Further details on the design are reported elsewhere [13].

### Setting

Thirty-three primary care practices in London (n = 18) and the North-West of England (NW) (n = 15) took part in the study. Thirty-two practices covered 3 LTCs and one practice covered 2 LTCs. Ten practices provided patients for asthma (5 in London and 5 in NW), 16 for COPD (8 in London and 8 in NW), 10 for diabetes (5 in London and 5 in NW), 23 for epilepsy (13 in London and 10 in NW), 20 for heart failure (11 in London and 9 in NW) and 19 for stroke (12 in London and 7 in NW). The number of practices per LTC varied according to LTC prevalence and practice size (12 were small (<5800 patients), 13 medium (5800–10,500 patients) and 8 large (>10,500 patients)). A slightly larger number of practices was recruited from more deprived areas (Table 1).

**Table 1:** **Number of practices per social deprivation quintile**

	**Quintile**	Range (IMD rank 2010*)	**London**	**NW**	**Total**
**Most deprived**	**Q1**	1 – 6496	4	4	8
	**Q2**	6497 - 12992	4	6	10
	**Q3**	12993 - 19488	5	1	6
	**Q4**	19489 - 25984	4	2	6
**Least deprived**	**Q5**	25985 - 32482	1	2	3

*Indices of Multiple Deprivation 2010.

### Participants

Eligible patients were identified through an automatic and remote search of practices’ clinical systems. The search was conducted by a subcontracted IT company prior to the baseline survey and aimed to identify approximately 50% of the patients for each LTC in every practice based on odd or even months of patients’ birthdays. The search was based on Read codes in line with the criteria used in the Quality and Outcomes Framework (QOF) with two exceptions: patients with diabetes needed to be 18 years of age and patients with transient ischaemic attack(s) (TIAs) were excluded from the stroke group. Patients were invited into the survey for one LTC only; if they had multiple LTCs they were included for their rarest condition. The list of patients identified from the search was reviewed by a member of staff (usually a GP or a nurse) to exclude any patients who were not considered suitable to be invited into the study. The instruction to practices was to exclude patients if invitation into the survey might cause serious distress.

### Patient-reported outcome measures (PROMs)

Both a generic PROM and appropriate disease-specific PROMs were included in the surveys, as well as standard demographics questions and a question on comorbidities. The follow-up survey also included a ‘change in health’ question to ascertain how much respondents believed their health had changed over the last year (i.e. since the administration of the baseline survey). The ‘change in health’ question was rated on a five-point scale (much better, a little better, about the same, a little worse and much worse).

The generic PROM was the EQ-5D [13] which is a measure of health status primarily designed to provide a single-index value which represents the utility of specific health states. It comprises 5 items (one each on mobility, self-care, usual activities, pain/discomfort, and anxiety/depression) and takes approximately five minutes to complete. Items are scored on a three-point scale and a single-index value is calculated typically with a score range from 1 (perfect health) to 0 (death). Scores below 0 can be obtained indicating states worse than death. The EQ-5D also includes a Visual Analogue Scale (VAS), ranging from 0 ‘worst imaginable health state’ to 100 ‘best imaginable health state’.

The disease-specific PROMs were the mini Asthma Quality of Life Questionnaire (MiniAQLQ) [14,15], the Clinical COPD Questionnaire (CCQ) [16,17], the Diabetes Health Profile (DHP) [18,19] Quality of Life in Epilepsy (QOLIE-31) [20], Minnesota Living with Heart Failure Questionnaire (MLHFQ) [21,22] and the Stroke Impact Scale version 3 (SIS) [23]. Table 2 presents details on the number of items, dimensions and scoring of each of the disease-specific PROMs. Evidence for their use in the UK was available, apart for the SIS. The PROMs were selected on the basis of their psychometric properties evaluated by review work (full details of the psychometric properties can be found in the review for each LTC on this web-page http://phi.uhce.ox.ac.uk/newpubs.php, accessed 13.12.12) and licensing agreements. Licenses could be secured for the psychometrically strongest PROMs for 4 LTCs (asthma, epilepsy, heart failure and stroke). In COPD and diabetes, licenses could not be secured and consequently the PROMs identified as second best, in terms of their psychometric properties, were selected for these LTCs.

**Table 2:** **Description of the disease-specific PROMs**

**PROM/ Country of development**	**Dimensions (n items)**	**Score range**
**Mini Asthma Quality of Life Questionnaire (mini-AQLQ) [** 14 **,** 15 **]/Canada**	Total score (all 15 items), Activity limitations (4 items) , Symptoms (5 items), Emotional function (3 items), Environmental stimuli (3 items)	1-7 where 1 is ‘severe impairment’ and 7 is ‘no impairment’
**Clinical COPD questionnaire (CCQ) [** 16 **,** 17 **]/The Netherlands**	Total score (all 10 items), Symptoms (4 items), Functional state (4 items), Mental state (2 items)	0-6 where 0 ‘very good health status’ and 6 ‘extremely poor health status’
**Diabetes Health Profile (DHP) [** 18 **,** 19 **]/United Kingdom**	Psychological distress (6 items), Barriers to activity (7 items), Disinhibited eating (5 items)	0-100 with a higher score representing higher dysfunction
**Quality of Life in Epilepsy Inventory (QOLIE) [** 20 **]/United States** NB we used 30 items as the Visual Analogue Scale was not included	Total score (all 30 items), Overall quality of life (2 items), Seizure/ worry (5 items), Emotional well-being (5 items), Energy/ fatigue (4 items), Cognitive (6 items), Medication effects (3 items), Social function (5 items)	0-100 with higher scores reflecting better quality of life
**Minnesota Living with Heart Failure Questionnaire (MLHFQ) [** 21 **,** 22 **]/United States**	Total score (all 21 items), Physical dimension (8 items), Emotional dimension (5 items)	Total score 0–105, physical dimension score 0–40 and emotional dimension score 0–25. On all dimensions a higher score means more impairment
**Stroke Impact Scale (SIS) [** 23 **]/United States**	Strength (4 items), Memory (7 items), Emotion (9 items), Communication (7 items), Activities of Daily Living (ADL) (10 items), Mobility (9 items), Hand function (5 items), Handicap (8 items) , Physical dimension (hand function, strength, mobility and ADL, 28 items)	0-6 where 0 ‘very good health status’ and 6 ‘extremely poor health status’

### Analysis

The data were entered into SPSS version 18 and verified by a professional data entry company. Data are only presented for participants who completed both the baseline and follow-up questionnaires. Descriptive statistics were used to describe the sample, PROMs scores at baseline and follow-up and the ‘change in health’ question. The ‘change in health’ question was recoded into improvement (i.e. much better and a little better), stable (i.e. about the same) and deterioration (i.e. a little worse and much worse). Levels of missing data for individual PROMs items and PROM dimensions were assessed. No data imputation was performed; therefore the sample size in the analyses may be lower than the number of respondents. Changes in the PROMs scores for respondents to both the baseline and follow-up surveys were assessed with paired t-tests. The relationship between PROMs change scores (=follow-up scores- baseline scores) and self-reported change in health (i.e. improved, stable or deteriorated) was assessed by analysis of variance and the significant results are presented. Regression analysis was used to assess any differences in mean changes scores by participant age (age category 18–44 years served as reference category), gender, time since diagnosis of the LTC and number of comorbidities. The level of significance was set at p < 0.05.

## Results

The baseline achieved a 38.4% response rate (n = 1721, asthma n = 395 (30.0%), COPD n = 279 (49.2%), diabetes n = 448 (40.0%), epilepsy n = 180 (34.0%), heart failure n = 262 (50.0%) and stroke n = 152 (36.4%)). A total of 1603 (93.1%) baseline participants consented to be followed up. The response rate at follow-up was 71.5% (n = 1136, asthma n = 267 (73.0%), COPD n = 187 (71.4%), diabetes n = 321 (75.7%), epilepsy n = 104 (62.7%), heart failure n = 155 (66.2%) and stroke n = 102 (74.5%)). Response rate was significantly different at follow-up by LTC (p = 0.015), age (p < 0.001), ethnicity (p = 0.008) and region (p = 0.007), with epilepsy and heart failure patients being less likely to respond than patients with one of the other LTCs, as were younger patients, those based in London and those from ethnic minority backgrounds. The baseline mean EQ-5D score was significantly lower (p < 0.001) in non-responders to follow-up (mean 0.66, SD 0.33) than in follow-up responders (mean 0.73, SD 0.29). Of the 1136 responders, 52.7% were male and 47.3% female (p < 0.001) and 78.1% were aged 55 years or above. Age differed significantly by disease, with asthma and epilepsy including a larger proportion of younger respondents (p < 0.001). Details of the response rates have been previously published [13].

### Data quality

The rates of missing data for the baseline and follow-up surveys are presented in Table 3. The rates of missing data are presented as a range i.e. the item/dimension with the lowest rate of missing data to the item/dimension with the highest rate of missing data. A change score was computed between the baseline and follow-up and missing data rates for the change score are also presented. Overall the rate of missing data was slightly higher at follow up than at baseline. However, missing data rates were low for the EQ-5D and slightly higher, although still acceptable, for the EQ-5D VAS. The Mini-AQLQ (asthma), the CCQ (COPD) and the DHP (diabetes) also had little missing data, although the cumulative effect of missing data meant that the rate of missing data of the change score between baseline and follow up was >10% for some dimensions. Rates of missing data were >10% for some items and dimensions of the QOLIE (epilepsy), the MLHFQ (heart failure) and SIS (stroke), leading to high rates of missing data on the change score.

**Table 3:** **Rates (%) of missing data for the EQ-5D and disease-specific PROMs for the baseline and follow-up surveys**

		**Individual Items**			**Dimensions**			
**LTC**	**PROM**	**N**	**% missing baseline**	**% missing follow-up**	**N**	**% missing baseline**	**% missing follow-up**	**% missing change score**
**Asthma**	Mini-AQLQ	15	0-3.8	0.8-6.8	5	1.1-7.1	3.4-11.7	4.1-15.0
	EQ-5D	5	0.4-0.8	0.8-1.9	1	1.1	3.0	4.1
	EQ-5D VAS	1	3.4	3.8	N/A	N/A	N/A	6.8
**COPD**	CCQ	10	1.6-3.7	0-3.7	4	4.3-9.1	2.1-8.6	5.9-14.4
	EQ-5D	5	0-1.1	1.1-2.1	1	2.1	4.8	5.3
	EQ-5D VAS	1	5.0	4.4	N/A	N/A	N/A	7.8
**Diabetes**	DHP	18	0-2.8	1.2-3.1	3	1.6-6.2	2.8-7.5	4.4-12.1
	EQ-5D	5	0.9-2.5	1.6-2.2	1	3.4	3.7	6.2
	EQ-5D VAS	1	5.0	4.4	N/A	N/A	N/A	7.8
**Epilepsy**	QOLIE	31	0-27.9	1.0-30.8	8	0-42.3	2.9-50.0	2.9-61.5
	EQ-5D	5	0-1.9	1.0-4.8	1	1.9	6.7	8.7
	EQ-5D VAS	1	9.6	5.8	N/A	N/A	N/A	12.5
**Heart failure**	MLHFQ	21	0.6-11.0	3.2-21.3	3	5.8-28.4	9.0-36.1	14.8-48.4
	EQ-5D	5	0-3.9	1.9-4.5	1	4.5	7.7	11.6
	EQ-5D VAS	1	2.6	5.2	N/A	N/A	N/A	6.5
**Stroke**	SIS	60	2.9-23.5	2.0-22.5	10	5.9-36.3	5.9-38.2	9.8-52.9
	EQ-5D	5	1.0-3.9	2.0-4.9	1	3.9	5.8	8.8
	EQ-5D VAS	1	11.8	10.8	N/A	N/A	N/A	19.6

### PROMs scores

The mean PROMs scores for all LTCs are presented in Table 4, together with the change scores. The only significant change for the EQ-5D was found for the Visual Analogue Scale (VAS) in heart failure between baseline and follow-up, and for the ‘change in health’ question. Significant differences between baseline and follow-up scores were found on the disease-specific PROMs for 1 (of 4) asthma dimension and 1 (of 9) stroke dimension. No significant differences were found for the 4 COPD, 3 diabetes, 7 epilepsy and 3 heart failure dimensions of disease-specific measures.

**Table 4:** **PROMs scores and change by LTC**

**N**		**Baseline mean**	**Follow-up mean**	**Mean change**	**Lower CI (95%)**	**Upper CI (95%)**	**p**
**Asthma EQ-5D**							
**York A1 tariff**	255	0.83	0.84	0.01	0.00	0.02	0.23
**VAS**	248	73.77	74.33	0.56	−1.09	2.21	0.50
**Asthma MINI-AQLQ**							
**Symptoms**	252	5.29	5.29	0.00	−0.12	0.12	0.99
**Activity Limitations**	240	6.08	5.92	−0.15	−0.26	−0.05	0.00
**Emotional Functioning**	253	5.37	5.28	−0.09	−0.23	0.05	0.21
**Environmental Stimuli**	255	5.30	5.24	−0.05	−0.17	0.07	0.38
**Total QOL**	226	5.60	5.52	−0.07	−0.17	0.02	0.13
**N**		**Baseline mean**	**Follow-up mean**	**Mean change**	**Lower CI (95%)**	**Upper CI (95%)**	**p**
**COPD EQ-5D**							
**York A1 tariff**	177	0.67	0.67	0.00	−0.02	0.01	0.77
**VAS**	173	62.29	62.14	−0.14	−2.94	2.66	0.92
**COPD CCQ**							
**Symptoms**	171	2.60	2.60	0.01	−0.14	0.16	0.92
**Functional State**	176	2.03	2.14	0.11	−0.03	0.25	0.12
**Mental State**	174	2.11	2.20	0.10	−0.09	0.29	0.32
**Total QOL**	160	2.22	2.28	0.06	−0.07	0.19	0.38
**N**		**Baseline mean**	**Follow-up mean**	**Mean change**	**Lower CI (95%)**	**Upper CI (95%)**	**p**
**Diabetes EQ-5D**							
**York A1 tariff**	301	0.73	0.72	0.00	−0.01	0.01	0.60
**VAS**	296	68.16	69.76	1.60	−0.19	3.39	0.08
**Diabetes DHP**							
**Psychological distress**	301	16.35	16.59	0.24	−1.09	1.57	0.72
**Barriers to activity**	282	22.17	22.39	0.22	−1.22	1.66	0.76
**Disinhibited eating**	307	30.16	30.08	−0.09	−1.71	1.54	0.92
**N**		**Baseline mean**	**Follow-up mean**	**Mean change**	**Lower CI (95%)**	**Upper CI (95%)**	**p**
**Epilepsy EQ-5D**							
**York A1 tariff**	95	0.76	0.76	0.00	−0.02	0.02	0.89
**VAS**	91	71.40	73.59	2.20	−0.96	5.36	0.17
**Epilepsy QOLIE**							
**Seizure worry**	95	64.49	65.32	0.82	−3.05	4.69	0.67
**Overall QOL**	79	68.26	68.58	0.32	−2.79	3.42	0.84
**Emotional well-being**	95	67.58	67.24	−0.34	−3.69	3.02	0.84
**Energy**	98	54.34	51.99	−2.35	−5.18	0.49	0.10
**Cognitive**	89	63.92	64.69	0.77	−2.18	3.73	0.60
**Medication effects**	98	62.59	61.65	−0.94	−5.67	3.80	0.70
**Social function**	53	79.25	79.04	−0.21	−5.14	4.73	0.93
**Total QOL**	40	69.92	70.13	0.22	−2.93	3.36	0.89
**N**		**Baseline mean**	**Follow-up mean**	**Mean change**	**Lower CI (95%)**	**Upper CI (95%)**	**p**
**Heart failure EQ-5D**							
**York A1 tariff**	137	0.64	0.64	0.00	−0.02	0.01	0.62
**VAS**	145	62.20	58.67	−3.53	−6.69	−0.38	0.03
**Heart failure MLHFQ**							
**Total QOL**	80	36.91	35.10	−1.81	−4.46	0.84	0.18
**Physical dimension**	125	18.44	18.02	−0.42	−1.62	0.77	0.48
**Emotional dimension**	132	7.79	7.48	−0.31	−0.99	0.36	0.36
**N**		**Baseline mean**	**Follow-up mean**	**Mean change**	**Lower CI (95%)**	**Upper CI (95%)**	**p**
**Stroke EQ-5D**							
**York A1 tariff**	93	0.67	0.67	−0.01	−0.03	0.01	0.45
**VAS**	82	73.84	71.96	−1.88	−5.12	1.37	0.25
**Stroke SIS**							
**Strength**	72	66.75	65.97	−0.78	−5.22	3.66	0.73
**Hand function**	76	73.22	72.17	−1.05	−3.67	1.57	0.43
**Mobility**	79	78.83	76.72	−2.11	−4.46	0.24	0.08
**Memory**	91	81.32	81.04	−0.27	−3.23	2.68	0.85
**ADL**	80	82.22	79.66	−2.56	−4.48	−0.65	0.01
**Communication**	92	86.88	85.05	−1.82	−4.26	0.61	0.14
**Emotion**	77	72.08	71.90	−0.18	−3.56	3.20	0.92
**Handicap**	48	72.20	72.92	0.72	−3.78	5.21	0.75
**Physical dimension**	52	78.08	76.35	−1.72	−3.67	0.23	0.08

In response to the ‘change in health’ question, approximately half of the respondents reported their health to have stayed stable in the last year, with approximately a quarter reporting deterioration and another quarter reporting improvement (Table 5). Reported change in health differed significantly (p < 0.001) between conditions, with COPD and heart failure respondents being more likely to report deterioration and stroke and epilepsy patients more likely to report improvement. Despite about half the respondents reporting a change, a significant relationship between health change and disease-specific PROMs scores was found on all 4 asthma dimensions, on all four COPD and 2 (out of 9) stroke dimensions. Table 6 reports the significant changes, and the full set of results including the non-significant findings are provided as a supplementary file (Additional file 1: Table S1).

**Table 5:** **Self-reported health change after one year**

	**Improvement**		**Stable**		**Deterioration**	
	**n**	**%**	**n**	**%**	**n**	**%**
**Asthma**	70	26.7	142	54.2	50	19.1
**COPD**	30	16.5	79	43.4	73	40.1
**Diabetes**	73	23.9	181	59.3	51	16.7
**Epilepsy**	30	30.0	58	58.0	12	12.0
**Heart failure**	24	15.7	76	49.7	53	34.6
**Stroke**	35	35.4	48	48.5	16	16.2
**Total**	262	23.8	584	53.0	255	23.2

**Table 6:** **Significant relationships between PROMs mean change scores and self-reported ‘change in health’**

**PROM dimensions**	**Change in health**	**N**	**Mean change score**	**95% CI**		**p**
				**Lower**	**Upper**	
**Asthma**						
Symptoms	Improvement	66	0.42	0.23	0.62	<0.001
	Stable	136	−0.03	−0.17	0.12	
	Deterioration	48	−0.52	−0.85	−0.18	
Activity Limitations	Improvement	66	0.12	−0.05	0.29	<0.001
	Stable	131	−0.11	−0.23	0.01	
	Deterioration	41	−0.74	−1.07	−0.40	
Emotional Functioning	Improvement	68	0.32	0.06	0.58	<0.001
	Stable	136	−0.11	−0.28	0.06	
	Deterioration	47	−0.60	−0.98	−0.22	
Environmental Stimuli	Improvement	67	0.29	0.06	0.53	<0.001
	Stable	139	−0.12	−0.28	0.03	
	Deterioration	46	−0.35	−0.65	−0.04	
Total Quality of Life	Improvement	62	0.27	0.10	0.43	<0.001
	Stable	126	−0.09	−0.21	0.02	
	Deterioration	36	−0.60	−0.88	−0.32	
**COPD**						
Symptoms	Improvement	30	−0.34	−0.77	0.09	0.03
	Stable	72	−0.05	−0.26	0.16	
	Deterioration	66	0.22	−0.03	0.47	
Mental	Improvement	28	−0.50	−0.99	−0.01	<0.001
	Stable	73	−0.01	−0.27	0.26	
	Deterioration	69	0.41	0.08	0.73	
Functional State	Improvement	29	−0.41	−0.73	−0.10	0.001
	Stable	73	0.07	−0.08	0.22	
	Deterioration	70	0.33	0.07	0.60	
Total QOL	Improvement	28	−0.42	−0.75	−0.08	<0.001
	Stable	66	−0.02	−0.17	0.13	
	Deterioration	63	0.32	0.09	0.55	
**Stroke**						
Hand Function	Improvement	27	1.11	−2.06	4.28	0.048
	Stable	37	−0.27	−4.35	3.81	
	Deterioration	12	−8.33	−16.94	0.27	
ADL	Improvement	29	0.00	−1.82	1.82	0.042
	Stable	38	−3.03	−6.09	0.04	
	Deterioration	12	−7.29	−14.76	0.17	

Regression analysis examining demographic factors (age and gender) and health factors (time since diagnosis of LTC and number of comorbidities) found only three significant relationships across all the PROMs change scores – diabetes psychological distress dimension (adjusted R square = 0.042, p = 0.008); stroke EQ-5D York Tariff (adjusted R square =0.11, p = 0.024) and stroke memory dimension (adjusted R square 0.23, P < 0.001). In diabetes, participants with a higher number of comorbidities were more likely to report change in psychological distress (p < 0.001). In stroke, men were less likely to report change on the EQ-5D than women (p = 0.002); and on the stroke memory dimension younger people were less likely to report change than the other 3 age groups (all p < 0.001) and people with a higher number of comorbidities were more likely to report change (p = 0.015).

## Discussion

PROMs are increasingly becoming part of the NHS for monitoring LTCs and quality of life (outcomes). PROMs may be an important tool in outcome assessment in any health care system and changes over time could in principle be analysed in relation to health care services received. There are few longitudinal studies in a population-based sample on the change of health in LTCs. In this study, patients, with a confirmed diagnosis of one of six LTCs, completed a generic and a disease-specific PROM at two points in time one year apart. The cohort design aimed to provide evidence of whether there was intra-individual change in health status over one year. The survey included LTCs which might be expected to vary in their trajectories over time; the natural history of conditions such as COPD and heart failure being likely to decline more rapidly compared to conditions such as asthma and epilepsy which may be expected to be more stable over long periods of time.

Differences between cohort baseline and follow-up PROMs scores were found for single sub-scales in the disease-specific PROMs for asthma and stroke, and the EQ-5D VAS for heart failure. For the majority of other PROMs scores for these three conditions and for all scales of COPD, diabetes and, epilepsy no changes were observed over one year. It could be argued that even the few significant changes observed may have arisen due to multiple testing. This means that overall health-related quality of life of all six conditions appeared stable over the course of one year. There may be non-response bias, as between 24.3% (diabetes) and 37.3% (epilepsy) of baseline respondents did not return the follow-up questionnaire. It may be possible that respondents who deteriorated were less likely to participate in the follow-up. Indeed participants who scored lower on the EQ-5D at baseline were less likely to respond to the follow-up. It should also be highlighted that the response rate to the baseline survey was 38%, although this is similar to other surveys in primary care samples [24-26]. Response to a survey is more likely when the questionnaire is shorter [27] and there may be fewer missing data meaning it becomes feasible to calculate a score as suggested for the SIS [28]. It may be possible that non-responders to the baseline had lower health status, similarly to the non-responders to the follow-up. In elective surgery, people who were less well pre-operatively were less likely to complete PROMs [29].

No data imputation was performed for several reasons. For the EQ-5D and three of the disease-specific PROMs (asthma, diabetes and COPD) the rate of missing data was negligible and therefore data imputation was not necessary. Data imputation methods were not specified by the developers of the PROMs for the QOLIE (epilepsy) and the MLHFQ (heart failure). Stroke had clear guidance on data imputation methods and a high rate of missing data on some items; and consequently data imputation was performed for stroke but imputation did not make a significant difference to the findings (details available in the full report [30]). The main reason to not impute the data was the primary goal of the study, i.e. the study aimed to evaluate the feasibility of collecting PROMs data in LTCs, including the degree of completion of the administered PROMs. Therefore the rate of missing data is considered a finding in itself and is another indicator as to the suitability of these PROMs for the collection of PROMs data for LTCs through primary care.

It is possible that the absence of change over time in some of the LTCs is due to the PROMs used not being responsive enough to detect change. There is broad agreement on the psychometric properties that a PROM should demonstrate [31]. Using PROMs to monitor outcomes in LTCs can only be successful if PROMs are able to detect health change or in other words the PROMs are responsive to change. It seems unlikely that the PROMs used in this study are not responsive to change as generally, disease-specific instruments are more likely to be sensitive and therefore more responsive to change in comparison to generic measures. In this study disease-specific PROMs were more likely than the generic EQ-5D to detect a change in the case of asthma and COPD, when compared to patients’ retrospective judgements of health change of the last year. Extensive review work summarised evidence on responsiveness for the majority of the disease-specific PROMs including the MiniAQLQ for asthma [32], the DHP for diabetes [33], the QOLIE-31 for epilepsy [34] and the MHLFQ for heart failure [35]. The PROMs used had been selected on the basis of extensive review work and responsiveness was one amongst a range of psychometric criteria considered in their selection. At the time of the reviews, information on responsiveness was available for the majority of the instruments; however the evidence was more mixed and/or limited for COPD (CCQ) [36] and stroke (SIS) [37]. The EQ-5D has been shown in previous studies to be responsive to change, although either the time periods for follow-up were longer [38,39], participants were at a more advanced stage of disease at the time of the study [40,41], participants were hospitalized [42] or had been given a drug intervention [43].

For all LTCs, in response to a simple retrospective health change question about their LTC compared to a year before, substantial proportions of respondents reported improvement (23.8%) or deterioration (23.2%), with individuals with COPD (40%) particularly likely to notice deterioration, compared with respondents with diabetes and epilepsy who were more likely to view their condition as stable (59.3% and 58.0% respectively). Stroke respondents were the most likely to have experienced improvement (35.4%). The majority of the respondents reported their health to have stayed stable over the period of one year. This may be an indication that a period of one year is not long enough to identify changes in health status. Given the more limited scope for improvement compared with the dramatic improvement in health status observed via PROMs for elective orthopaedic surgical procedures, more work is needed to gain evidence on the rate of deterioration and trajectory (such as slow vs. rapid deterioration, periods of stability and exacerbations) and to identify health changes that are meaningful for PROMs for LTCs in diagnosed validated samples. To our knowledge, there is currently little evidence via condition-specific PROMs for primary care.

## Conclusions

Few significant differences were found between the baseline and follow up surveys or between self-reported change in health and PROMs scores. This could be explained by the time frame of one year being too short for change to occur or by the PROMs not being responsive enough to change in a primary care sample. Currently, little is known about speed of progression of various LTCs particularly in relation to health-related quality of life. If PROMs are to be used routinely to monitor outcomes in LTCs, further research is needed to ensure that the PROMs used are reliable, valid and sensitive enough to detect change and that the time frame for data collection is appropriate for change to have occurred.

## Additional file

Additional file 1: Table S1. Relationships between mean change in PROM scores and self-reported ‘change in health’ question.

## Abbreviations

PROM: Patient-reported outcome measure
LTC: Long-term condition
NHS: National health service
ANOVA: Analysis of variance
MiniAQLQ: Mini asthma quality of life questionnaire
CCQ: Clinical COPD questionnaire
DHP: Diabetes health profile
QOLIE: Quality of life in epilepsy inventory
MLHFQ: Minnesota living with heart failure questionnaire
SIS: Stroke impact scale
