# Supplementary material for: Change in health status in long-term conditions over a one year period: a cohort survey using patient-reported outcome measures
Source: Health Qual Life Outcomes. 2014 Aug 12;12:123. doi: 10.1186/s12955-014-0123-2 (PMC4243951; doi:10.1186/s12955-014-0123-2)
Supplement: Additional file 1: Table S1. — Relationships between mean change in PROM scores and self-reported ‘change in health’ question. [file 12955_2014_123_MOESM1_ESM.docx]

Table 6 (supplementary file) Relationships between mean change in PROM scores and self-reported ‘change in health’ question

| **LTC** | **PROM dimensions** | **Change in Health** | **N** | **Mean change score** | **95% CI** | | **p** |
| --- | --- | --- | --- | --- | --- | --- | --- |
|  |  |  |  |  | **Lower** | **Upper** |  |
| Asthma | EQ5D York Tariff | Improvement | 69 | 0.02 | -0.01 | 0.04 | 0.41 |
|  |  | Stable | 137 | 0.00 | -0.01 | 0.01 |  |
|  |  | Deterioration | 47 | 0.01 | -0.02 | 0.04 |  |
|  | EQ5D VAS | Improvement | 64 | 0.89 | -2.53 | 4.31 | 0.39 |
|  |  | Stable | 132 | 0.91 | -1.20 | 3.02 |  |
|  |  | Deterioration | 49 | -1.90 | -5.53 | 1.74 |  |
|  | Symptoms | Improvement | 66 | 0.42 | 0.23 | 0.62 | 0.00 |
|  |  | Stable | 136 | -0.03 | -0.17 | 0.12 |  |
|  |  | Deterioration | 48 | -0.52 | -0.85 | -0.18 |  |
|  | Activity Limitations | Improvement | 66 | 0.12 | -0.05 | 0.29 | 0.00 |
|  |  | Stable | 131 | -0.11 | -0.23 | 0.01 |  |
|  |  | Deterioration | 41 | -0.74 | -1.07 | -0.40 |  |
|  | Emotional Functioning | Improvement | 68 | 0.32 | 0.06 | 0.58 | 0.00 |
|  |  | Stable | 136 | -0.11 | -0.28 | 0.06 |  |
|  |  | Deterioration | 47 | -0.60 | -0.98 | -0.22 |  |
|  | Environmental Stimuli | Improvement | 67 | 0.29 | 0.06 | 0.53 | 0.00 |
|  |  | Stable | 139 | -0.12 | -0.28 | 0.03 |  |
|  |  | Deterioration | 46 | -0.35 | -0.65 | -0.04 |  |
|  | Total Quality of Life | Improvement | 62 | 0.27 | 0.10 | 0.43 | 0.00 |
|  |  | Stable | 126 | -0.09 | -0.21 | 0.02 |  |
|  |  | Deterioration | 36 | -0.60 | -0.88 | -0.32 |  |
| **LTC** | **PROM dimensions** | **Change in Health** | **N** | **Mean change score** | **95% CI** | | **p** |
|  |  |  |  |  | **Lower** | **Upper** |  |
| COPD | EQ5D York Tariff | Improvement | 28 | 0.02 | -0.03 | 0.07 | 0.23 |
|  |  | Stable | 75 | 0.01 | -0.01 | 0.02 |  |
|  |  | Deterioration | 70 | -0.02 | -0.05 | 0.01 |  |
|  | EQ5D VAS | Improvement | 30 | 0.30 | -8.67 | 9.27 | 0.10 |
|  |  | Stable | 73 | 3.18 | 0.34 | 6.02 |  |
|  |  | Deterioration | 66 | -3.61 | -8.93 | 1.71 |  |
|  | Symptoms | Improvement | 30 | -0.34 | -0.77 | 0.09 | 0.03 |
|  |  | Stable | 72 | -0.05 | -0.26 | 0.16 |  |
|  |  | Deterioration | 66 | 0.22 | -0.03 | 0.47 |  |
|  | Mental | Improvement | 28 | -0.50 | -0.99 | -0.01 | 0.00 |
|  |  | Stable | 73 | -0.01 | -0.27 | 0.26 |  |
|  |  | Deterioration | 69 | 0.41 | 0.08 | 0.73 |  |
|  | Functional State | Improvement | 29 | -0.41 | -0.73 | -0.10 | 0.00 |
|  |  | Stable | 73 | 0.07 | -0.08 | 0.22 |  |
|  |  | Deterioration | 70 | 0.33 | 0.07 | 0.60 |  |
|  | Total QOL | Improvement | 28 | -0.42 | -0.75 | -0.08 | 0.00 |
|  |  | Stable | 66 | -0.02 | -0.17 | 0.13 |  |
|  |  | Deterioration | 63 | 0.32 | 0.09 | 0.55 |  |
| **LTC** | **PROM dimensions** | **Change in Health** | **N** | **Mean change score** | **95% CI** | | **p** |
|  |  |  |  |  | **Lower** | **Upper** |  |
| Diabetes | EQ5D York Tariff | Improvement | 67 | 0.00 | -0.02 | 0.03 | 0.73 |
|  |  | Stable | 175 | -0.01 | -0.02 | 0.01 |  |
|  |  | Deterioration | 48 | 0.00 | -0.03 | 0.04 |  |
|  | EQ5D VAS | Improvement | 67 | 2.46 | -1.07 | 6.00 | 0.62 |
|  |  | Stable | 171 | 1.48 | -0.98 | 3.94 |  |
|  |  | Deterioration | 49 | -0.39 | -4.59 | 3.81 |  |
|  | Psychological Distress | Improvement | 66 | 1.09 | -2.00 | 4.19 | 0.61 |
|  |  | Stable | 172 | -0.55 | -2.20 | 1.10 |  |
|  |  | Deterioration | 49 | -0.68 | -4.73 | 3.37 |  |
|  | Disinhibited Eating | Improvement | 70 | 0.19 | -3.26 | 3.65 | 0.75 |
|  |  | Stable | 175 | -0.15 | -2.32 | 2.02 |  |
|  |  | Deterioration | 49 | -1.77 | -6.17 | 2.63 |  |
|  | Barriers to Activities | Improvement | 62 | 0.61 | -2.77 | 4.00 | 0.85 |
|  |  | Stable | 160 | -0.18 | -1.81 | 1.46 |  |
|  |  | Deterioration | 47 | 0.81 | -4.07 | 5.69 |  |
| **LTC** | **PROM dimensions** | **Change in Health** | **N** | **Mean change score** | **95% CI** | | **p** |
|  |  |  |  |  | **Lower** | **Upper** |  |
| Epilepsy | EQ5D York Tariff | Improvement | 28 | 0.01 | -0.02 | 0.04 | 0.58 |
|  |  | Stable | 53 | 0.00 | -0.03 | 0.02 |  |
|  |  | Deterioration | 12 | -0.02 | -0.11 | 0.07 |  |
|  | EQ5D VAS | Improvement | 25 | 4.68 | -0.05 | 9.41 | 0.69 |
|  |  | Stable | 54 | 1.76 | -2.21 | 5.73 |  |
|  |  | Deterioration | 10 | 0.80 | -17.38 | 18.98 |  |
|  | Seizure Worry | Improvement | 26 | 1.53 | -6.52 | 9.58 | 0.85 |
|  |  | Stable | 53 | 0.28 | -5.13 | 5.69 |  |
|  |  | Deterioration | 12 | 3.72 | -7.63 | 15.07 |  |
|  | Overall QOL | Improvement | 20 | -0.13 | -5.75 | 5.50 | 0.76 |
|  |  | Stable | 50 | 0.60 | -3.13 | 4.33 |  |
|  |  | Deterioration | 7 | 4.29 | -16.60 | 25.17 |  |
|  | Emotional Well-being | Improvement | 27 | -0.74 | -7.22 | 5.74 | 0.90 |
|  |  | Stable | 55 | 0.07 | -4.45 | 4.60 |  |
|  |  | Deterioration | 9 | 2.22 | -12.04 | 16.49 |  |
|  | Cognitive | Improvement | 22 | 3.50 | -3.26 | 10.25 | 0.33 |
|  |  | Stable | 52 | 0.07 | -3.60 | 3.75 |  |
|  |  | Deterioration | 11 | -4.17 | -14.40 | 6.07 |  |
|  | Medication Effects | Improvement | 28 | -2.08 | -13.01 | 8.84 | 0.76 |
|  |  | Stable | 55 | 0.45 | -5.17 | 6.08 |  |
|  |  | Deterioration | 12 | -4.86 | -23.07 | 13.34 |  |
|  | Social Function | Improvement | 12 | -4.08 | -15.88 | 7.71 | 0.29 |
|  |  | Stable | 37 | 2.41 | -3.51 | 8.32 |  |
|  |  | Deterioration | 2 | -14.00 | -141.06 | 113.06 |  |
|  | Energy | Improvement | 27 | -3.33 | -9.06 | 2.39 | 0.50 |
|  |  | Stable | 56 | -1.16 | -5.14 | 2.82 |  |
|  |  | Deterioration | 12 | -6.25 | -13.31 | 0.81 |  |
|  | Total QOL | Improvement | 8 | 0.47 | -7.77 | 8.72 | 0.58 |
|  |  | Stable | 30 | 0.75 | -3.00 | 4.50 |  |
|  |  | Deterioration | 1 | -9.86 |  |  |  |
| **LTC** | **PROM dimensions** | **Change in Health** | **N** | **Mean change score** | **95% CI** | | **p** |
|  |  |  |  |  | **Lower** | **Upper** |  |
| Heart Failure | EQ5D York Tariff | Improvement | 20 | -0.01 | -0.06 | 0.04 | 0.96 |
|  |  | Stable | 68 | 0.00 | -0.02 | 0.02 |  |
|  |  | Deterioration | 48 | -0.01 | -0.04 | 0.03 |  |
|  | EQ5D VAS | Improvement | 22 | 6.45 | -0.27 | 13.18 | 0.02 |
|  |  | Stable | 71 | -5.34 | -9.59 | -1.09 |  |
|  |  | Deterioration | 51 | -3.69 | -8.78 | 1.41 |  |
|  | Total QOL | Improvement | 9 | -5.00 | -12.41 | 2.41 | 0.40 |
|  |  | Stable | 46 | -2.52 | -6.61 | 1.56 |  |
|  |  | Deterioration | 25 | 0.64 | -2.82 | 4.10 |  |
|  | Physical Dimension | Improvement | 19 | 1.84 | -0.73 | 4.41 | 0.19 |
|  |  | Stable | 61 | 1.03 | -0.84 | 2.91 |  |
|  |  | Deterioration | 45 | -1.00 | -2.91 | 0.91 |  |
|  | Emotional Dimension | Improvement | 19 | -0.68 | -2.24 | 0.87 | 0.42 |
|  |  | Stable | 66 | 0.65 | -0.42 | 1.72 |  |
|  |  | Deterioration | 46 | 0.24 | -0.81 | 1.29 |  |
| **LTC** | **PROM dimensions** | **Change in Health** | **N** | **Mean change score** | **95% CI** | | **p** |
|  |  |  |  |  | **Lower** | **Upper** |  |
| Stroke | EQ5D York Tariff | Improvement | 33 | 0.01 | -0.01 | 0.03 | 0.38 |
|  |  | Stable | 44 | -0.02 | -0.06 | 0.01 |  |
|  |  | Deterioration | 13 | -0.02 | -0.10 | 0.06 |  |
|  | EQ5D VAS | Improvement | 30 | -1.27 | -6.20 | 3.67 | 0.58 |
|  |  | Stable | 38 | -3.32 | -7.40 | 0.77 |  |
|  |  | Deterioration | 12 | 1.67 | -13.43 | 16.76 |  |
|  | Strength | Improvement | 24 | 3.65 | -3.00 | 10.29 | 0.33 |
|  |  | Stable | 37 | -2.20 | -7.23 | 2.84 |  |
|  |  | Deterioration | 11 | -5.68 | -27.62 | 16.25 |  |
|  | Hand Function | Improvement | 27 | 1.11 | -2.06 | 4.28 | 0.05 |
|  |  | Stable | 37 | -0.27 | -4.35 | 3.81 |  |
|  |  | Deterioration | 12 | -8.33 | -16.94 | 0.27 |  |
|  | Memory | Improvement | 34 | -0.42 | -3.60 | 2.76 | 0.76 |
|  |  | Stable | 43 | -0.91 | -5.66 | 3.83 |  |
|  |  | Deterioration | 13 | 2.47 | -10.15 | 15.10 |  |
|  | Mobility | Improvement | 32 | 0.00 | -2.79 | 2.79 | 0.16 |
|  |  | Stable | 38 | -3.22 | -7.13 | 0.70 |  |
|  |  | Deterioration | 8 | -7.29 | -16.58 | 1.99 |  |
|  | ADL | Improvement | 29 | 0.00 | -1.82 | 1.82 | 0.04 |
|  |  | Stable | 38 | -3.03 | -6.09 | 0.04 |  |
|  |  | Deterioration | 12 | -7.29 | -14.76 | 0.17 |  |
|  | Communication | Improvement | 34 | -0.21 | -3.07 | 2.65 | 0.32 |
|  |  | Stable | 44 | -1.95 | -5.90 | 2.01 |  |
|  |  | Deterioration | 13 | -6.04 | -15.22 | 3.13 |  |
|  | Emotion | Improvement | 30 | -0.93 | -7.37 | 5.52 | 0.87 |
|  |  | Stable | 35 | 0.63 | -4.03 | 5.30 |  |
|  |  | Deterioration | 11 | 1.52 | -7.60 | 10.63 |  |
|  | Handicap | Improvement | 16 | 6.25 | -3.05 | 15.55 | 0.21 |
|  |  | Stable | 27 | -1.74 | -6.79 | 3.32 |  |
|  |  | Deterioration | 5 | -3.75 | -29.25 | 21.75 |  |
|  | Physical Dimension | Improvement | 19 | 0.37 | -2.85 | 3.59 | 0.14 |
|  |  | Stable | 29 | -3.44 | -6.15 | -0.74 |  |
|  |  | Deterioration | 4 | 0.79 | -7.79 | 9.37 |  |
